# Granuloma after the Injection of Poly-D,L-Lactic Acid (PDLLA) Treated with Triamcinolone

**DOI:** 10.1155/2024/6544506

**Published:** 2024-04-25

**Authors:** Kateryn Michelle Perez Willis, Rosario Ramirez Galvez

**Affiliations:** ^1^Department of Dermatology, Derma Laser KPW Medical Center, Lima, Peru; ^2^Department of Aesthetic Medicine, Ricardo Palma Medical Center, Lima, Peru

## Abstract

Biostimulators are the latest trends in cosmetic procedures, substances such as PDLLA are used to induce collagen synthesis by a subclinical inflammatory reaction. We are describing a granuloma-like reaction case presentation 4 months after the application of PDLLA and its complete resolution with injections of triamcinolone. A 45-year-old female with any past medical history of allergies or immune diseases was injected with PDLLA on the mandibular border and cheek area to correct skin laxity. Four months after the application, the patient reported facial edema and granuloma-like reactions according to clinical examination on all the application areas. The ultrasound reports showed the presence of multiple nodules in the injection areas; therefore, we decided to apply triamcinolone to the granulomatous reaction areas 2 times a month and Prednisone 20 mg daily for 3 days followed by 10 mg for 2 days. After 4 applications, the adverse reaction was completely solved. Biostimulators are biocompatible and resorbable substances; however, nodules and/or granulomas have been reported as rare adverse events. Intralesional and oral steroids can allow us to treat this kind of adverse events.

## 1. Introduction

Dermal fillers such as hyaluronic acid have been widely used for decades in cosmetic procedures, lately, patients are choosing biostimulators instead of hyaluronic acid fillers seeking for the “natural” stimulation of collagen instead of the filling effect of the hyaluronic acid products [1, 2]. Biostimulators such as poly-l-lactic Acid (PLLA), poly-D,L-lactic acid (PDLLA) induce collagen synthesis stimulating Fibroblasts by causing a subclinical inflammatory reaction [3–5]. These substances are absorbable and biocompatible within the tissue; however, some rare adverse events have been described after using collagen inductors, nodules and granulomas. We must emphasize in this two different types of reactions since they have different causes and clinical presentations [3, 4].

A granuloma is caused mainly by an exaggerated inflammatory reaction of the host; its clinical presentation has a very important edema and big hard granulomatous reaction that can be noticeable and palpable all over the injections were applied, and the granuloma should be confirmed by a histopathologic evaluation [4, 5]. Its onset time can be between 6 to 24 months after injection; it can grow to the size of a bean or bigger; and it can be accompanied by skin discoloration and edema; and reacts well to intralesional steroid treatment [4].

A nodule is caused mainly by an anomalous accumulation of the product due to a bad technique of application or its accumulation in dynamic facial muscles (example: orbicularis oris), its clinical presentation has well defined nodules that are can be noticeable or not depending on the location (neck, hands, or forehead area) are noticeable areas [4, 5]. Its time of onset is 1 to 2 months after application and the nodules are usually solitaire, with proximity to dynamic muscles, the size can be of a lentil, a pea or bigger; It may or may not react well with intralesional steroids and in rare cases excisions are necessary [4].

When injecting a biostimulator we can't predict the immune response of our patients, therefore it is important that we manage a protocol that can guide us to treat this type of reactions.

## 2. Case Report

**Figure 1:**
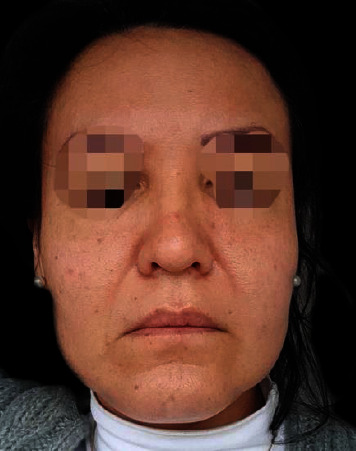
Patient face with edema all over the application areas, granulomas on the sides of the face are present showing a square heavy face.

A 45-year-old female with any past medical history of allergies or immune disease presented to our Aesthetic Department after 4 months of PDLLA application complaining according to clinical evaluation of facial edema and granuloma like formations all over the injection areas of the PDLLA (Figure 1).

She had never experienced any complication with Hyaluronic acid fillers, botulinum toxin or threads. 2 weeks prior to the clinical presentation of the adverse reaction she had injected intravenously Ascorbic Acid 15 mg and Iron due to low Hemoglobin.

**Figure 2:**
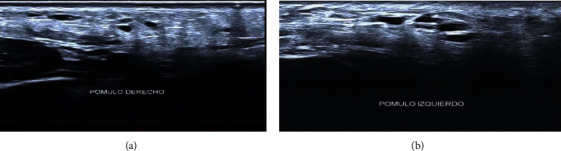
Ultrasound performed with a 24 MHz high-frequency linear transducer, high-resolution Doppler; (a, b), respectively.

After talking a through history from our patient, it was proved that she did not suffer from any infection or trauma in that area. Furthermore, she had not undergone any dental or medical procedures after the PDLLA application and has no medical history or any recent Covid 19 vaccination. The only related new treatment and the clinical presentation of the reaction was the Vitamin C and Iron IV injection. The ultrasound reports the presence of hyper refringent material with defined edges at the subcutaneous cellular plane located in the malar and lower mandibular region on both sides where PDLLA was applied (Figure 2).

**Figure 3:**
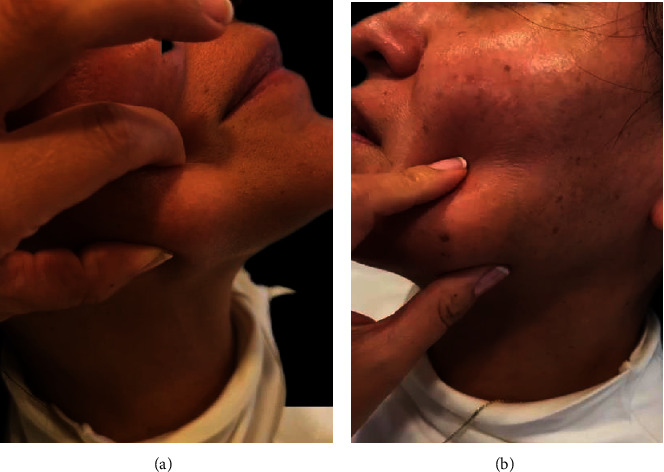
Palpation of the granulomas on the sides of the face, on the palpation it felt stone like firmness on 2 gigantic granulomas with the size of 8 cm × 4 cm approximately, (a, b), respectively.

Physical examination revealed multiple granulomas like formations with stone-like firmness in palpation located all over the injection area of PDLLA, the application site was the border of the jaw, (jawline, both sides) and the cheeks. Edema was present all over the application of PDLLA area and she described itchiness all over the face (Figure 3).

**Figure 4:**
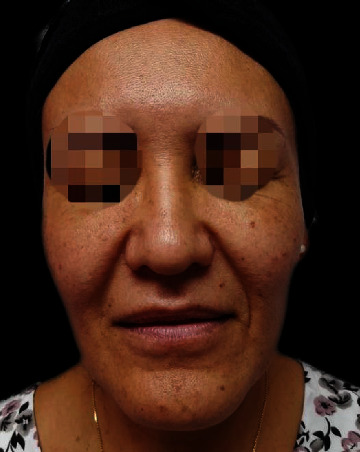
Granuloma was completely solved after the application of intralesional triamcinolone every 15 days, a total of 4 applications were needed.

After receiving the patient it was decided to initiate Prednisone 20 mg daily for 3 days, and she responded to the treatment, however, when she stopped taking the prednisone the edema and the hardness rebound immediately so we decided to inject intralesional triamcinolone in one side and Collagenase in the other side to compare which treatment would have a better and rapid outcome, the triamcinolone side responded a lot better compared to collagenase so only treat the granuloma with triamcinolone every 2 weeks until its complete resolution, was injected triamcinolone 4 times total with successful results (Figure 4).

## 3. Discussion

Collagen stimulation is a common cosmetic treatment increasing in popularity, PDLLA is a new molecule that due to the high porosity property of the microspheres the total volume of all PDLLA microspheres is several folds higher than PLLA microparticles [4, 6].

As a result, PDLLA microspheres can not only stimulate neo-tissues formation but also provide scaffolds for them. The neo-tissues first grow on the surface of these microspheres, then they grow more to fill the interspace of microspheres. At the same time, these porous microspheres degrade gradually while neo-tissues grow inside microspheres. The original volume of these microsphere scaffolds is replaced by neo-tissues and remains the same in the following months. PDLLA and PLLA are similar molecules, but these molecules are not free of complications, short-term adverse reactions to PLLA injections reported in the literature include pain, edema, bleeding, ecchymosis, dyschromia, overcorrection [6].

These side effects usually occur within days after injection and resolve spontaneously within 1 to 2 weeks. Other type of complications such us embolism, and localized cellulitis have been also described and need a specific treatment for each case. PLLA-induced granulomas and nodules have been described as late onset complications that need a specific and longer treatment, these complications can be disfiguring for our patients and can present suddenly after months of application without any specific reason. Similar complications have been reported more often with hyaluronic acid due to covid vaccines. Further studies need to be done to find the cause that targets the formation of granulomas with different substances that are widely used in cosmetic procedures for facial or body rejuvenation [4].

Treatment options for late-onset subcutaneous nodules and granulomas include intralesional steroids, systemic steroids, systemic antibiotics, intense pulsed light, 5-fluorouracil, allopurinol, and surgical removal [4], In this case, intralesional triamcinolone (20 mg/ml) injection had positive responses and the treatment was well tolerated compared to Collagenase injections. There are some reported cases only of PLLA filler induced late-onset foreign body granulomatous reaction in Europe and America thus we strongly suggest that all the colleagues that apply Biostimulators and any other injectables must manage a protocol for early and late onset complications to give a successful and rapid solution for our patients. It is important to highlight that now there are no published data between the comparison of the safety profile between PLLA and PDLLA, PDLLA claims to be safer because of the isomerization of the molecule; however, there is no scientific report to proof this. In this case, there was no histopathological confirmation for the diagnosis of granulomas; however, the clinical signs and symptoms, and ultrasound were crucial in defining the treatment rate. In this case, the patient was satisfied with the resolution of the problem for which she came for consultation.

## Data Availability

Data sharing not applicable to this article as no datasets were generated or analyzed during the current study.
